# Bovine Peripheral Blood Derived Lymphocyte Proteome and Secretome Show Divergent Reaction of Bovine Immune Phenotypes after Stimulation with Pokeweed Mitogen

**DOI:** 10.3390/proteomes10010007

**Published:** 2022-02-08

**Authors:** Kristina J. H. Kleinwort, Roxane L. Degroote, Sieglinde Hirmer, Lucia Korbonits, Lea Lorenz, Armin M. Scholz, Stefanie M. Hauck, Cornelia A. Deeg

**Affiliations:** 1 Department of Veterinary Sciences, LMU Munich, D-82152 Planegg, Germany; k.kleinwort@lmu.de (K.J.H.K.); r.degroote@tiph.vetmed.uni-muenchen.de (R.L.D.); sieglinde.hirmer@tiph.vetmed.uni-muenchen.de (S.H.); lucia.korbonits@tiph.vetmed.uni-muenchen.de (L.K.); lea.lorenz@tiph.vetmed.uni-muenchen.de (L.L.); 2 Livestock Center of the Faculty of Veterinary Medicine, LMU Munich, D-85764 Oberschleißheim, Germany; armin.scholz@lvg.vetmed.uni-muenchen.de; 3 Research Unit Protein Science, Helmholtz Center Munich, German Research Center for Environmental Health, D-80939 Munich, Germany; hauck@helmholtz-muenchen.de

**Keywords:** Pokeweed mitogen, polyclonal cell stimulation, hyperproliferation, differential immune proteomes, deviant bovine immune phenotypes, quantitative label-free LC-MS/MS, cow

## Abstract

We recently identified a deviant bovine immune phenotype characterized by hyperproliferation of lymphocytes after polyclonal stimulation. This phenotype was first discovered in dams that responded to PregSure BVD vaccination by producing pathological antibodies, triggering the fatal disease “bovine neonatal pancytopenia” in calves. The aim of the study was to gain deeper insights into molecular processes occurring in lymphocytes of immune phenotypes and the effect on their secretome after immune stimulation. Two discovery proteomic experiments were performed with unstimulated and Pokeweed Mitogen (PWM) stimulated lymphocytes, using label-free LC-MS/MS. In lymphocytes, 2447 proteins were quantified, and 1204 proteins were quantified in the secretome. Quantitative proteome analysis of immune deviant and control samples after PWM stimulation revealed clear differences. The increase in abundance of IL17A, IL17F, IL8, CCL5, LRRC59, and CLIC4 was higher in controls through mitogenic stimulation. In contrast, the abundance of IFNγ, IL2, IL2RA, CD83, and CD200 increased significantly more in immune deviant lymphocytes. Additional pathway enrichment analysis of differentially secreted proteins also yielded fundamental differences between the immune phenotypes. Our study provides a comprehensive dataset, which gives novel insights into proteome changes of lymphocytes from different bovine immune phenotypes. These differences point to the development of diverse immune responses of bovine immune phenotypes after immune stimulation.

## 1. Introduction

With a first occurrence in 2005, bovine neonatal pancytopenia (BNP) was a novel, fatal, vaccination-associated disease characterized by symptoms of hemorrhagic diathesis in newborn calves with a high mortality rate [1]. A novel vaccine against bovine viral diarrhea (PregSure BVD) induced fatal immune reactions with the production of alloreactive antibodies [2,3] in 5–10% of vaccinated cows [1]. These alloantibodies were transferred via colostrum and caused BNP in calves [1,2,4]. In our recent studies, peripheral blood mononuclear cells (PBMC) of BNP alloantibody-producing cows (BNP cows) reacted as significantly hyperproliferative after in vitro stimulation with different polyclonal mitogens, such as Pokeweed mitogen (PWM), Concanavalin A (ConA), and Banana lectin (BanLec), compared to cows who did not produce pathological alloantibodies after PregSure BVD vaccination (control cows) [5]. Furthermore, we detected this hyperproliferative response pattern in cows that were never vaccinated with PregSure BVD. Thus, we reasoned that this aberrant immune phenotype is present throughout the cattle population and termed it the immune deviant (ID) phenotype [5]. Previously, we completed extensive differential proteome analysis of lymphocytes from both phenotypes stimulated with T and B cell mitogen PWM [6], focusing on the differential expression of transcription factors [5]. Thus, the abundance of Signal Transducer and Activator of Transcription 3 and 5A increased significantly more in PBMC of ID cows after PWM stimulation compared to control PBMC [5]. As we were also interested in changes of the entire proteome, we analyzed this proteomic dataset in-depth to further compare the immunological reactions of both phenotypes on in vitro immune stimulation with PWM. Furthermore, we performed a secretome experiment using label-free LC–MS/MS. The aim was to analyze differences in proteins secreted by lymphocytes before and after in vitro stimulation with PWM between the bovine immune phenotypes. Using this hypothesis-generating proteomics approach, our aim was to better characterize the immune phenotypes of cows by gaining deeper insights into the molecular processes occurring in lymphocytes and the effect on their secretome after immune stimulation.

## 2. Materials and Methods

### 2.1. Animals

In this study, peripheral blood mononuclear cells (PBMC; lymphocytes and monocytes) of 13 cows were analyzed. For the mass spectrometric analysis of bovine PBMC samples, cells from four PregSure BVD vaccinated cows were examined. Two were PregSure BVD vaccinated control cows (*n* = 2), which responded inconspicuously to vaccination and did not produce BNP-inducing alloantibodies. Two were confirmed BNP donors (*n* = 2) vaccinated with PregSure BVD that responded aberrantly to vaccination by producing alloreactive antibodies, which induced BNP in their calves, confirmed by post-mortem analysis [5,7]. Samples of BNP donors are cited as immune deviant (ID) samples in the further course of the paper, as previous studies showed that the BNP immune phenotype is also present in PregSure BVD unvaccinated cows and was therefore called the ID phenotype in general [5]. All four PregSure BVD vaccinated cows used for mass spectrometric analysis came from one dairy farm, had the same breed, and were similar in age and parity. The basic vaccination of the dams with PregSure BVD was performed in 2007–2008. The sampling and the examination of the lymphocyte proteome for the present study was carried out in 2014, four years after PregSure BVD had been withdrawn from the European market [1,8]. At this timepoint, the number of living PregSure BVD vaccinated cows had already substantially declined, so we were only able to analyze fresh PBMC of two animals per phenotype. For the mass spectrometric analysis of the secretome samples, only PBMC from one of the PregSure BVD vaccinated control (*n* = 1) and one of the BNP cows (*n* = 1) could be examined, as the experiment was performed one year later, and only these PregSure BVD vaccinated cows were still available. For verification of differential IL2RA and CD83 expression in Western blot analysis, PBMC lysates from six control cows (*n* = 6), whereof one was a PregSure BVD vaccinated control cow, and PBMC lysates from five ID cows (*n* = 5), whereof two were PregSure BVD vaccinated BNP donors, were examined. The PregSure BVD unvaccinated control and ID cows also came from one dairy farm, had the same breed, and were similar in age and parity. The ID status of unvaccinated cows was determined by hyperproliferation in proliferation assays (technical replicates per cow ≥ 11). Withdrawal of bovine venous whole blood and experimental protocols were approved by the local authority Regierung von Oberbayern, Munich, permit no. ROB-55.2-2532.Vet_03-17-106. No experimental animals were used in this study. The study is reported in accordance with the ARRIVE guidelines. The permission from the dairy farm to use the blood samples from their animals for study purposes was obtained.

### 2.2. PBMC Preparation

Bovine venous whole blood was collected in sodium–heparin (25.000 I.U., ratiopharm, Ulm, Germany)-coated tubes, and PBMC were prepared as previously described [5]. Briefly, blood was diluted 1:2 in PBS (NaCl 136.9 mM (Sigma-Aldrich, part of Merck-Millipore, Darmstadt, Germany), Na_2_HPO_4_ × 2H_2_O 8.1 mM (AppliChem, Darmstadt, Germany), KH_2_PO_4_ 1.4 mM (Merck-Millipore, Darmstadt, Germany), and KCl 2.6 mM (Sigma-Aldrich); pH 7.2), and PBMC were isolated by density gradient centrifugation (room temperature, 290× *g*, 25 min, brake off) using Pancoll separating solution (PanBiotech, Aidenbach, Germany). PBMC were obtained from the intermediate phase, washed twice in PBS, and immediately used for in vitro stimulation. 

### 2.3. Polyclonal Stimulation of PBMC

After resuspension in RPMI 1640 (PanBiotech) with 10% fetal bovine serum (FBS, Biochrom, part of Merck Millipore, Darmstadt, Germany) and 1% Penicillin–Streptomycin (PanBiotech), bovine PBMC (2.2 × 10^7^ cells) were stimulated with Pokeweed mitogen (PWM, 5 µg/mL; Sigma-Aldrich) at 37 °C and 5% CO_2_, and unstimulated controls were incubated under the same conditions. After 48 h, cells were washed twice with PBS, supernatants were discarded, and PBMC were immediately fractionated before proteomic analysis. For the secretome dataset, samples were prepared equally, except that serum-free RPMI was used for cell incubation to avoid protein overlaps from the FBS in the mass spectrometric analysis. All secretome samples were centrifuged (1000× *g*) after 48 h of incubation; the supernatants were collected and subsequently digested in a filter-aided sample preparation (FASP). For control of proliferation rate of the bovine PBMC, proliferation assays were performed simultaneously in both the cellular and secretome approaches. Therefore, PBMC (1 × 10^5^ cells/well) were stimulated in triplicates for 48 h with 5 µg/mL PWM in 96-well plates as described [5]. After 34 h of stimulation, cells were pulsed for 14 h with 0.05 mCi/well (methyl-3H)-thymidine (Perkin Elmer, Hamburg, Germany) and harvested, and counts per minute were measured. The proliferation rate after stimulation was expressed as a factor of (3H)-thymidine incorporation in relation to the unstimulated cells. PBMC from PregSure BVD vaccinated BNP dams used in differential proteomics experiments, as well as PBMC from PregSure BVD unvaccinated ID animals used for Western blot experiments, were hyperproliferative as previously defined [5].

### 2.4. Subcellular Fractionation of PBMC

After stimulation, cells underwent subcellular fractionation for in-depth proteome analysis. Cells were separated into the cell surface fraction, nuclear fraction, and cytosolic fraction as previously described [9]. Briefly, surface proteins were labeled with biotin. After lysis, cytosolic and nuclear proteins were separated through differential centrifugation, while surface proteins were pulled down with Strep-Tactin Superflow Beads (IBA, Göttingen, Germany). Biotinylated and affinity-enriched surface proteins were digested with trypsin and PNGaseF directly on the streptavidin beads. Protein concentrations of the nuclear and cytosolic fractions were measured with a BCA protein assay [10], and 10 µg each were digested with Lys-C and trypsin in a FASP procedure.

### 2.5. Sample Digestion for Differential Proteome Analysis

Nuclear and cytosolic fractions of PBMC and secretome samples were digested by a modified FASP protocol as described [9,11]. Briefly, eluates were diluted 1:10 with 0.1 M tris/HCl (Sigma-Aldrich), pH 8.5, and 100 mM dithiothreitol (SERVA, Heidelberg, Germany) was added for 30 min at 60 °C. After cooling down, a UA buffer (8 M urea (Sigma-Aldrich) and 1 M tris-HCl, pH 8.5, diluted in HPLC-grade water) and 300 mM iodoacetamide (Merck-Millipore) were added and incubated for 30 min at room temperature in the dark. Eluates were transferred to 30 kDa cut-off centrifuge filters (Sartorius, Göttingen, Germany) and washed five times with UA-buffer and two times with ABC buffer (50 mM NH_3_HCO_3_ (Sigma-Aldrich) diluted in HPLC-grade water). After washing, proteins were subjected to proteolysis for 2 h at room temperature with 0.5 µg Lys C (Fujifilm Wako Chemicals Europe, Neuss, Germany) in an ABC-buffer, followed by the addition of 1 µg trypsin (Promega, Walldorf, Germany) and incubation at 37 °C overnight. Peptides were collected by centrifugation and acidified with 0.5% trifluoroacetic acid (Sigma-Aldrich).

### 2.6. Mass Spectrometric Analysis and Label-Free Quantification

Analysis of peptides was performed as described [5,12]. Acidified eluted peptides were analyzed in the data-dependent mode on a Q Exactive HF mass spectrometer (Thermo Fisher Scientific, Bremen, Germany) online coupled to an UItimate 3000 RSLC nano-HPLC (Dionex, Sunnyvale, CA, USA). Samples were automatically injected and loaded onto the C18 trap column, eluted after 5 min, and separated on the C18 analytical column (Acquity UPLC M-Class HSS T3 column, 1.8 µm, 75 µm × 250 mm; Waters) by a 90 min non-linear acetonitrile gradient at a flow rate of 250 nl/min. MS spectra were recorded at a resolution of 60,000, and after each MS1 cycle, the 10 most abundant peptide ions were selected for fragmentation. Acquired raw data was loaded into Progenesis QI software for proteomics for MS1 intensity-based label-free quantification (v2.5, Nonlinear Dynamics, Waters) and analyzed as described [9]. MS/MS spectra were exported and searched against the Ensembl bovine database (version 80) using the Mascot search engine (version 2.4.1; www.matrixscience.com). Search settings were enzyme trypsin, 10 ppm peptide mass tolerance and 0.02 Da fragment mass tolerance, one missed cleavage allowed, carbamidomethylation was set as a fixed modification, and methionine oxidation and asparagine and glutamine de-amidation were allowed as variable modifications. A Mascot-integrated decoy database search was performed with an average false discovery rate of <1%. Peptide assignments were re-imported into the Progenesis QI software. The abundances of all unique peptides allocated to each protein were summed up. The resulting normalized abundances of the individual proteins were used for the calculation of fold-changes of protein ratios between PWM stimulated and unstimulated samples.

### 2.7. Western Blots

A total of 1 × 10^7^ PBMC were lysed in lysis buffer (9 M urea, 2 M thiourea (Merck-Millipore), 65 mM dithiothreitol, 4% CHAPS (Carl Roth, Karlsruhe, Germany), and 5× Laemmli-buffer (250 mM tris-HCl, 5% SDS (AppliChem), 50% glycerol (SERVA), 20 mM 2-mercaptoethanol (Sigma-Aldrich), bromophenol blue (Sigma-Aldrich)) was added. Protein expression was analyzed separately for every biological replicate of controls (*n* = 6) and ID cases (*n* = 5), as well as for every technical replicate (*n* = 3). From each sample, 7 µg protein was separated by SDS-PAGE on 14% gels and blotted semidry onto PVDF membranes (GE Healthcare, Freiburg, Germany). To prevent unspecific binding, membranes were blocked with 4% PVP-T (Sigma-Aldrich). After washing, blots were incubated with a mouse anti-bovine CD25 (IL2RA) monoclonal antibody (clone IL-A111, Bio-Rad, Munich, Germany; 1:15,000), goat anti-bovine CD83 (goat polyclonal, Biorbyt, Cambridge, UK; 1:1000), and rabbit anti-beta Actin (rabbit polyclonal, Cell Signaling Technology, Darmstadt, Germany; 1:10,000) to control equal loading at 4 °C overnight. Secondary antibodies were taken respectively. POD-conjugated anti-mouse IgG antibody (Sigma-Aldrich; 1:5000), HRP-conjugated anti-goat IgG antibody (Thermo Fisher Scientific; 1:10,000), or HRP-conjugated anti-rabbit IgG (Fc) antibody (Bio-Rad; 1:10,000) were used for incubation at room temperature for one hour. After six washing steps, signaling was detected by enhanced chemoluminescence on an Amersham ImageQuant 800 biomolecular imager (GE Healthcare). Quantification of Western blot signals was achieved by using open source Image J software (https://imagej.net/ (accessed on 23 March 2021)) [13]. All abundances were normalized to respective beta Actin signals.

### 2.8. Data Analysis

Proteins with a PWM/unstimulated ratio in mass spectrometric analyses of at least 2 were considered differentially abundant. Statistical analysis of the cellular dataset was performed using Progenesis QI software (v3.0, Nonlinear Dynamics, Waters, Newcastle upon Tyne, UK). Transformed (“log-like” arcsinh(.)function) normalized abundances were used for one-way analysis of variance (ANOVA) calculations of all detected features. Values of *p* ≤ 0.05 indicated the statistical significance of the difference in group expression. A Venn diagram of differentially abundant proteins after stimulation was designed with Venny open source software (version 2.1.0, https://bioinfogp.cnb.csic.es/tools/venny/ (accessed on 4 November 2020)). Pathway enrichment analysis of cellular and secretome data was done with open source software ShinyGO (version 0.741, http://bioinformatics.sdstate.edu/go/ (accessed on 11 January 2022)) [14]. For determination of the Gaussian distribution of Western blot data, the Kolmogorow–Smirnow (KS) test was used. Since analyzed data showed no normal distribution (the KS test was not significant; *p* > 0.05), the Mann–Whitney test was further performed for statistical analysis. Statistical tests were performed using GraphPad Prism (version 5.04 for Windows, GraphPad Software, San Diego, CA, USA). Statistical probabilities were considered significant at *p* ≤ 0.05. For comparing IL2RA and CD83 expression changes in control and ID PBMC after PWM stimulation, unstimulated control and ID values were set to a factor of 1 ± SD.

### 2.9. Data Availability

The mass spectrometry proteomics data have been deposited to the ProteomeXchange Consortium via the PRIDE partner repository (https://www.ebi.ac.uk/pride/ (accessed on 31 January 2020 and 5 March 2020)) [15]. Data are available via identifiers PXD017350 (cellular dataset) and PXD017892 (secretome dataset).

## 3. Results

### 3.1. The Bovine Peripheral Blood Derived Lymphocyte Proteome Consists of 2447 Proteins

Using LC-MS/MS analysis, we identified the bovine whole lymphocyte proteome, comprising a total of 2447 proteins from all cell fractions (Table S1). The high number of identified proteins reflects the analytical depth due to high standard sample preparation and the latest analytical methods. Among the identified proteins, many immunological key proteins like 48 cluster of differentiation (CD) proteins [16] and cytokines [17] were present. 

### 3.2. The Proteome Shows Significant Differential Expression between Cow Immune Phenotypes after Polyclonal Immune Stimulation with Pokeweed Mitogen

**Figure 1:**
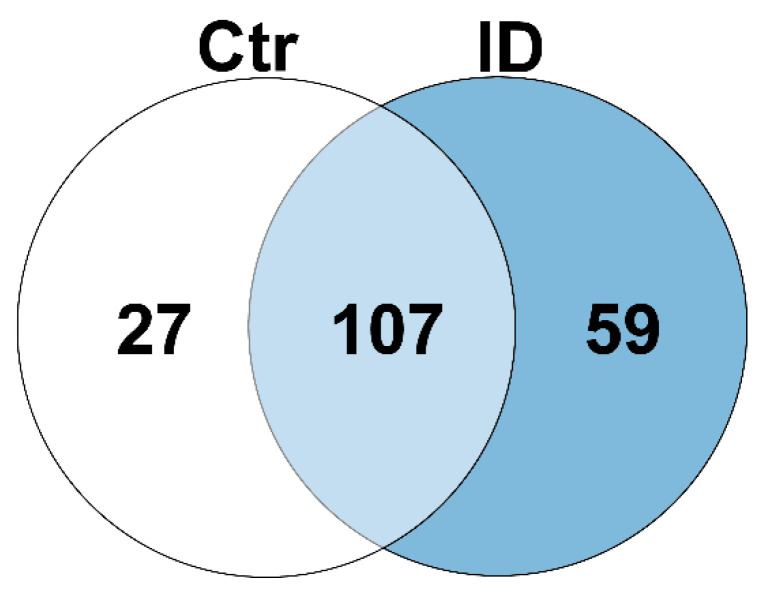
Significantly (*p* ≤ 0.05) differential proteins (ratio ≥ 2) in peripheral lymphocytes of control and ID cows after in vitro stimulation with PWM. In control PBMC (white and light blue), 134 proteins were differentially more abundant after PWM stimulation compared to unstimulated cells. In lymphocytes of ID cows (light blue and blue), 166 proteins were differentially more abundant after in vitro stimulation. Thereof, 107 proteins were identified in both control and ID PBMC (light blue).

After polyclonal stimulation with PWM, we found significant (*p* ≤ 0.05) changes in lymphocyte protein abundance (ratio cut-off PWM/unstim ≥ 2, number of unique peptides ≥ 2). Thus, in PWM stimulated control lymphocytes (*n* = 2), 134 proteins were differentially more abundant compared to unstimulated controls, and in lymphocytes of ID cows (*n* = 2), 166 proteins showed increased abundances after in vitro stimulation with PWM. Interestingly, 107 of these proteins showed increased abundance in both control and ID lymphocytes (Figure 1 and Table 1, light blue; Table S2), indicating a uniformly expressed group of canonical proteins in response to immune stimulation with PWM, regardless of immune phenotype (control/ID) of cows. Among the proteins exclusively increased in one phenotype after immune stimulation, 27 proteins were more abundant in control lymphocytes (Figure 1 and Table 1, white; Table S2), whereas 59 proteins showed higher abundance in ID lymphocytes (Figure 1 and Table 1, blue; Table S2).

In proteins exclusively more highly abundant in control lymphocytes, immunological key proteins were identified. Thus, Leucine-rich repeat-containing (LRRC) protein 59 as well as Chloride intracellular channel (CLIC) protein 4 were both 2.6-fold more abundant in stimulated control cells (Table 1, white; Table S2) compared to unstimulated controls. In contrast, other immunological key proteins were more abundant in ID lymphocytes after immune stimulation, amongst other solute carrier (SLC) transporters [18]. Although most of the identified members of the SLC family showed elevated abundance through stimulation in both immune phenotypes, the increase of abundance was clearly higher in lymphocytes of ID cows than in lymphocytes of controls. Overall, five SLC proteins (SLC1A5, SLC3A2, SLC1A4, SLC38A1, SLC29A1) were identified, with an increase of abundance of a maximum of 2.6-fold in control lymphocytes after PWM stimulation but at least a 7.5-fold increase in ID lymphocytes (Table 1, light blue; Table S2). Another important protein identified with a distinctly higher abundance in stimulated ID lymphocytes was interleukin-2 receptor subunit alpha (IL2RA). In stimulated ID lymphocytes, the abundance of IL2RA was 30.0-times higher than in unstimulated ID lymphocytes, whereas the increase in control cells was only 2.8-fold (Table 1, light blue; Table S2). Additionally, some CD antigens showed a clearly higher expression in lymphocytes from cows with an immune deviant phenotype after stimulation with PWM. Hence, CD83 expression in ID lymphocytes raised 86.8-fold after PWM stimulation compared to 2.8-fold in control lymphocytes (Table 1, light blue; Table S2), and CD200 was 6.9-times more abundant in ID lymphocytes after PWM stimulation compared to unstimulated ID cells (Table 1, blue; Table S2).

### 3.3. PWM Stimulation Leads to a Significantly Higher Expression of IL2RA and CD83 in ID Lymphocytes

**Figure 2:**
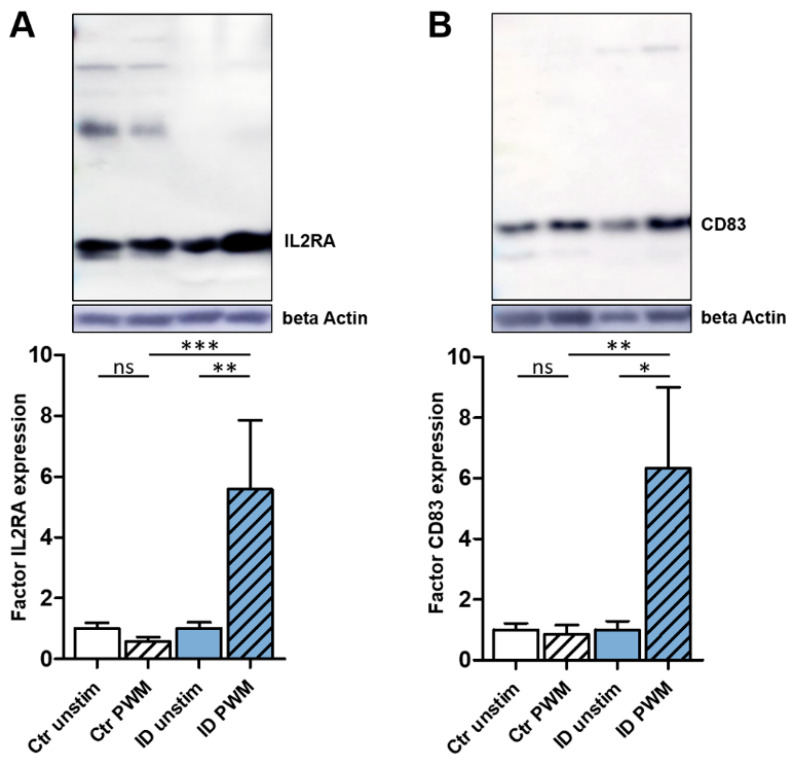
Western blot analysis of differential IL2RA and CD83 expression after PWM stimulation in PBMC of control and ID cows. Representative Western blots of IL2RA (**A**) and CD83 (**B**). (**A**) After PWM stimulation (PWM), Western blot analysis of the whole cell lysate from PBMC showed a 0.6-fold decreased IL2RA expression in control cells (white columns, *n* = 6) and a 5.6 fold-increased (** *p* ≤ 0.005) IL2RA expression in ID cells (blue columns, *n* = 5) compared to respective unstimulated PBMC (unstim, set to factor one). Direct comparison between PWM stimulated PBMC revealed a highly significant increase (*** *p* ≤ 0.0005) of IL2RA expression in ID cells. (**B**) After PWM stimulation (PWM), control PBMC (white columns, *n* = 6) showed a 0.9-fold decreased CD83 expression, and ID cells (blue columns, *n* = 5) showed a 6.3-fold increased (* *p* ≤ 0.05) CD83 expression compared to respective unstimulated PBMC (unstim, set to factor one). A direct comparison between PWM stimulated control and ID PBMC revealed a significant increase (** *p* ≤ 0.005) of CD83 expression. The corresponding Western blot membranes are shown in their entirety in Figure S1.

A significant increase of IL2RA and CD83 expression in ID lymphocytes through PWM stimulation was also shown by Western blot analysis (Figure 2). ID cells (*n* = 5) responded to PWM stimulation with a 5.6-fold higher expression of IL2RA compared to unstimulated ID cells (Figure 2A, blue columns; ** *p* ≤ 0.005), whereas IL2RA expression in control cells (*n* = 6) declined to a factor of 0.6 through PWM stimulation (Figure 2A, white columns). For CD83, the increase of expression in ID lymphocytes (*n* = 5) through PWM stimulation was also significant in Western blot analysis. Thus, ID cells expressed 6.3-times more CD83 after PWM stimulation (Figure 2B, blue columns; * *p* ≤ 0.05). In contrast, control lymphocytes (*n* = 6) showed almost no response to PWM stimulation regarding CD83 expression, with a decrease to a factor of 0.9 (Figure 2B, white columns). Thus, Western blot verified the clearly significant increase in the expression of IL2RA and CD83 for ID cells after PWM stimulation.

### 3.4. The Enriched Pathway Profile Reveals Functional Differences between the Lymphocytes of Bovine Immune Phenotypes after Immune Stimulation

**Figure 3:**
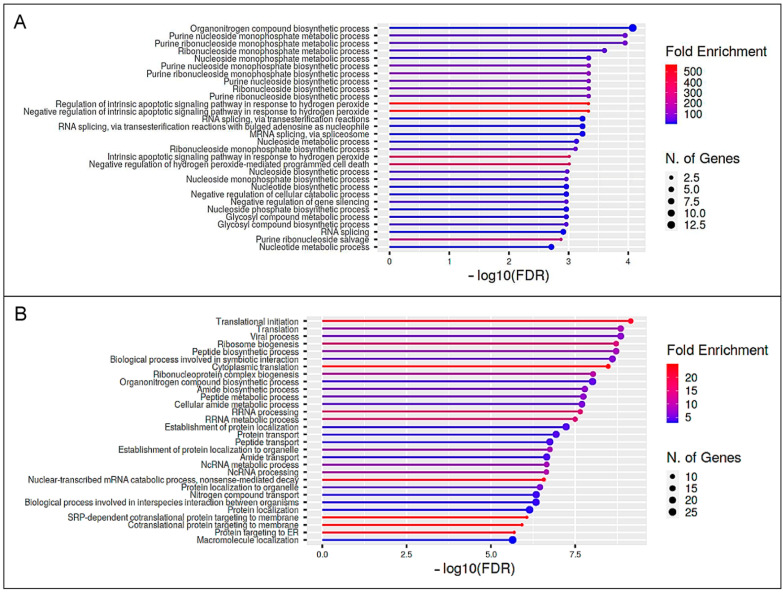
Pathway enrichment analysis of the cellular PBMC dataset. Functional enrichment showing the 30 most significant categories of biological processes (GO terms). Hierarchical clustering was performed with ShinyGO software v0.741 using human orthologue gene names of higher abundant PBMC proteins after PWM stimulation of lymphocytes from controls and immune deviant cows. The enrichment chart shows data for proteins that were exclusively more abundant in either controls (**A**) or immune deviant cows (**B**). The y-axis lists allocated pathways in order of the enrichment of FDR. The x-axis shows the FDR values for the enrichment of respective pathways. The color chart shows fold enrichment for each pathway. The size of dots corresponds to the number of genes assigned to each pathway. The corresponding pathway enrichment data is shown in more detail in Table S3.

As we were interested in the functional impact of the differential immune proteome of the bovine immune phenotypes, we further performed a pathway enrichment analysis using ShinyGO software. We compared pathways associated to exclusively higher abundant proteins expressed by control lymphocytes after PWM stimulation (Table 1, white; Table S2) with pathways allocated to exclusively higher abundant proteins in ID lymphocytes after stimulation (Table 1, blue; Table S2). In this study, clear differences between the bovine immune phenotypes became apparent, as the most enriched pathways (GO terms) significantly varied (Figure 3, Table S3). Interestingly, in ID lymphocytes, the pathway for “viral process” was significantly enriched after in vitro stimulation (Figure 3B, Table S3).

Considerably, additional analysis of pathways associated to higher abundant proteins expressed in both control and ID lymphocytes after PWM stimulation (Table 1, light blue; Table S2) revealed IL2RA as well as CD83 in the pathway for “response to stress” (Table S3).

### 3.5. The Bovine Peripheral Blood Lymphocyte Secretome Is Composed of 1204 Proteins with a Changed Cytokine Profile between Cow Immune Phenotypes

**Figure 4:**
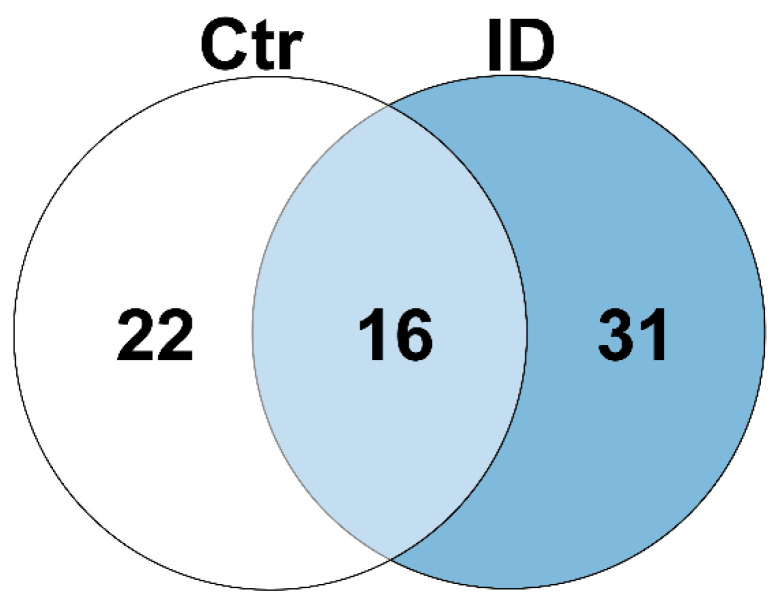
Differentially abundant proteins (ratio ≥ 2) after in vitro stimulation with PWM in the secretome of bovine immune phenotypes. In the secretome of control lymphocytes (white and light blue), 38 proteins were differentially more abundant after PWM stimulation compared to unstimulated cells. In the secretome of lymphocytes of ID cows (light blue and blue), 47 proteins were differentially more abundant after in vitro stimulation. Thereof, 16 proteins were identified in both the control and ID secretomes (light blue).

To expand our analyses on the impact of immune stimulation on lymphocytes, we subsequently performed a proteome analysis of bovine secretome samples. For this, we established a special FBS-free stimulation protocol to avoid the interference of proteins from a serum supplement in cell culture media in mass spectrometric measurements. In this in-depth proteome analysis, we identified a total of 1204 proteins in the secretome of bovine PBMC (Table S4), comprising 784 proteins that were identified with at least two unique peptides. Quantitative comparison of these proteins indicated differential secretion between bovine immune phenotypes after polyclonal stimulation. Although 16 proteins were more abundant in the secretome of both immune phenotypes after stimulation (Figure 4 and Table 2, light blue; Table S5), 22 proteins were exclusively more abundant in the control secretome (*n* = 1) after PWM stimulation compared to the secretome of unstimulated control lymphocytes (Figure 4 and Table 2, white; Table S5). In contrast, 31 secreted proteins showed increased abundances after in vitro stimulation of PBMC exclusively in the ID secretome (*n* = 1; Figure 4 and Table 2, blue; Table S5). 

Among the differentially abundant proteins, many cytokines as major regulators of innate and adaptive immunity [17] were detectable. Thus, interleukin (IL) 17A was 7.1-times more abundant after stimulation in the control secretome (1.4 in ID), and also, IL17F was 2.5-fold upregulated in controls (0.8 in ID) after PWM stimulation compared to their unstimulated secretome (Table 2, white; Table S5). Additionally, IL8 (CXCL8) was 6.2-times more abundantly secreted by PWM stimulated control lymphocytes than by unstimulated control cells (0.8 in ID) (Table 2, white; Table S5), and the chemokine (C-C motif) ligand (CCL) 5 was 36.4-fold more abundant (2.0 in ID; Table 2, light blue; Table S5). In contrast, interferon gamma (IFNγ) was clearly 177.6-fold upregulated from ID lymphocytes after PWM stimulation, whereas in control lymphocytes, secreted IFNγ was only 5.0-fold more abundant (IFNG, Table 2, light blue; Table S5). Furthermore, the abundance of IL2 was 30.5-fold higher in the secretome of ID cows after stimulation and, therefore, almost twice as much as in ID lymphocytes (17.3) (Table 2, light blue; Table S5).

### 3.6. The Enriched Pathway Profile Indicates a Functional Impact of Differentially Secreted Proteins of Bovine Immune Phenotypes after Immune Stimulation

**Figure 5:**
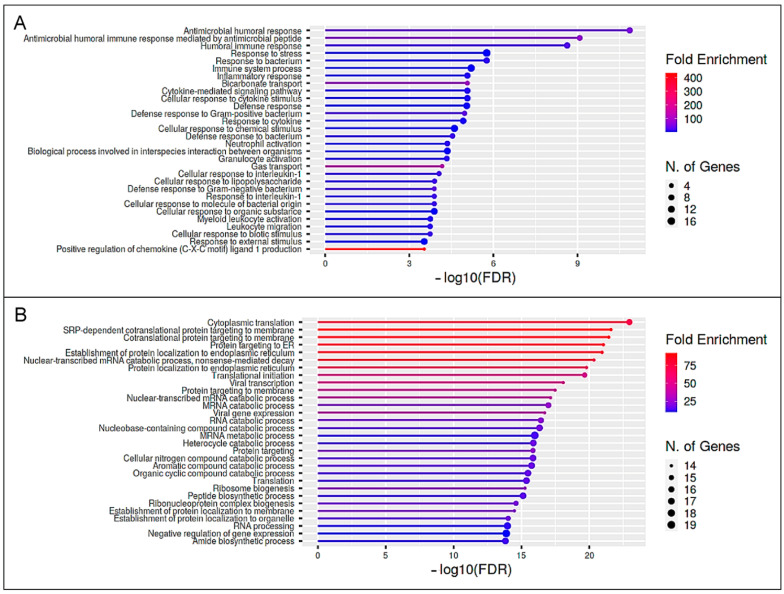
Pathway enrichment analysis of the secretome dataset. Functional enrichment showing the 30 most significant categories of biological processes (GO terms). Hierarchical clustering was performed with ShinyGO software v0.741 using human orthologue gene names of more abundant secretome proteins after PWM stimulation of lymphocytes from controls and immune deviant cows. The enrichment chart shows data for secretome proteins which were exclusively more abundant in either controls (**A**) or immune deviant cows (**B**). The y-axis lists allocated pathways in order of the enrichment of FDR. The x-axis shows FDR values for the enrichment of respective pathways. The color chart shows the fold enrichment for each pathway. The size of dots corresponds to the number of genes assigned to each pathway. The corresponding pathway enrichment data is shown in more detail in Table S6.

To further analyze the functional impact of the differential immune secretome of both bovine immune phenotypes after immune stimulation, we used ShinyGO software for the pathway enrichment analysis. We compared pathways associated with exclusively more abundant proteins secreted by control lymphocytes after PWM stimulation (Table 2, white; Table S5) with pathways allocated to exclusively more abundant proteins secreted by ID lymphocytes after stimulation (Table 2, blue; Table S5). Considering the most significant categories of biological processes (GO terms) of this study, clear differences in pathway enrichment became apparent (Figure 5, Table S6). Whereas most over-represented pathways in control cows were directly associated to the immune response to the stimulus (Figure 5A, Table S6), over-represented pathways in ID cows were not linked to immunological reactions but rather to protein biosynthesis and metabolic processes (Figure 5B, Table S6). Noticeably, over-represented pathways in the ID secretome mainly arise from an increased abundance of ribosomal proteins (Table S6). Thus, 14 of the 31 (45%) more abundant (ratio ≥ 2) serum proteins after PMW stimulation in ID cows belong to the family of ribosomal proteins. Since ribosomal proteins also possess ribosome-independent functions in immune signaling [19], the elevated abundance of these proteins is especially interesting. These data clearly underline the deviant reaction of both immune phenotypes to an immune stimulus in vitro, which may most likely also occur in vivo.

## 4. Discussion

Lymphocytes play crucial roles in the immune defense of hosts against pathogens [20]. The analysis of proteomic profiles of lymphocytes can reveal more information about their functions and the reactions to external immune stimuli [20]. With our study, we have provided a comprehensive proteomic characterization of bovine peripheral blood derived lymphocytes for two different bovine immune phenotypes. Using label-free LC-MS/MS, we generated two proteomic datasets for bovine peripheral lymphocytes and their respective secretomes. Since 5–10% of vaccinated cows showed an aberrant vaccination response and reacted with the production of fatal BNP-inducing antibodies to vaccination with PregSure BVD [1], we additionally aimed to examine the effect of immune stimulation with T and B cell mitogen PWM [6,21] on lymphocytes of both immune phenotypes. The T cell-dependent B cell activation process induced by PWM was found to be monocyte-dependent [21]. Thus, after PWM activation of human lymphocytes, the initial event for in vitro T lymphocyte blastogenic responses and B lymphocyte Ig secretion is the binding of PWM to sugar residues on the monocyte surface membrane, followed by lymphocyte interaction [21]. Furthermore, the T cell proliferation induced by PWM was shown to be IL2-dependent [22]. After in vitro stimulation with PWM, the lymphocytes of ID cows proliferated significantly stronger (4.5-fold) than cells from vaccinated control dams (ID to control, **** *p* < 0.0001) [5]. With an unprecedented high resolution of 2447 identified proteins in the cellular dataset (Table S1), we are, to our knowledge, the first to describe the bovine whole-lymphocyte proteome in such a high resolution. In the secretome dataset, we identified a total of 1204 proteins in the secretome of unstimulated and PWM-stimulated bovine lymphocytes (Table S4). After polyclonal stimulation with PWM, distinct changes in protein abundance compared to unstimulated cells became apparent between physiological and aberrant vaccination responders. 

Despite the small sample size, our data are of great interest and value, since our study depicts a discovery experiment using rare samples of vaccinated cows, which are no longer available. To ensure the highest possible degree of comparability, the samples for our label-free proteomic measurements came from cows of one dairy farm, which had the same breed and were similar in age and parity. To validate our findings, Western blot experiments were performed using a bigger sample size. Thereby, we were able to confirm the differential abundance of two proteins that were discovered in our proteomic approach, namely IL2RA and CD83. Thus, our study provides valid and novel data on bovine immune phenotypes. 

Focusing our analysis on proteins with immunological functions, LRRC59 was identified among differentially expressed proteins in the control lymphocytes (PWM/unstim ratio 2.6; Table 1, white; Table S2). LRRC59 is a positive regulator of type I IFN signaling, thereby provoking the comprehensive host defenses upon viral infections [23]. Type I IFNs have a broad range of biological functions, including modulation of innate and adaptive immune responses, anti-proliferative functions, and most importantly, antiviral activities [24]. Type I interferon signaling regulates primary CD4^+^ T cells, which are necessary for the generation of protective immune responses following vaccination or infection, and thereby determines host survival [25]. Hence, the higher expression of LRRC59 in control lymphocytes after PWM stimulation can provoke the comprehensive host’s defenses upon viral infections. 

Furthermore, CLIC4, which was 2.6-times more abundant in control lymphocytes after PWM stimulation (Table 1, white; Table S2), was shown to be required for an optimal macrophage response to diverse pathogens in mice [26]. Thus, stable murine macrophage lines overexpressing CLIC4 produced more TNF, IL6, IL12, and CCL5 than mock transfectants when exposed to LPS in vitro [26]. Interestingly, in our study, the bovine control lymphocytes also secreted much more CCL5 after PWM stimulation (PWM/unstim ratio 36.4; Table 2, light blue; Table S5) underlining this evidence. CCL5 is released by degranulation from activated virus-specific CD8^+^ T cells [27] and has been shown to induce the in vitro migration and recruitment of T cells, dendritic cells, eosinophils, natural killer cells, mast cells, and basophils [28]. Thus, CLIC4 knock-out mice responded to *Listeria monocytogenes* infection in vivo by producing less inflammatory cytokines and chemokines and were impaired in their ability to clear infection [26]. These data indicate CLIC4 as an important protein in innate immune responses to microbial products [26]. 

The higher expression of IL8 (CXCL8; PWM/unstim ratio 6.2; Table 2, white; Table S5) as a potent chemoattractant for granulocytes, inducing neutrophil recruitment and chemotaxis [29], confirms the upregulation of proteins associated with innate immune responses in control lymphocytes after PWM stimulation. Noticeably, the abundance of IL17A was also 7.1-times higher in the secretome of control lymphocytes after PWM stimulation, and the abundance of IL17F was 2.5-times higher (Table 2, white; Table S5). IL17 is mainly produced by Th17 cells [30] but also by several other cell types, like CD8^+^ T cells [31] and natural killer cells [32,33]. Th17 cells have been associated with many autoimmune diseases and extracellular pathogen infections [34,35]. However, several studies in recent years have demonstrated that Th17 cells and IL17 are also required for host defense against intracellular bacterial infection, such as *Salmonella* [36], *M. tuberculosis* [37,38], *Chlamydia* [39], and *L. monocytogenes* [40]. In summary, the higher abundance of mentioned proteins in lymphocytes from physiologically to vaccination-responding control cows after PWM stimulation indicates the development of a protective immune response of those cows after viral or other intracellular infections. 

Comparing higher abundant proteins in lymphocytes of aberrantly responder cows to vaccination after PWM stimulation, we found other proteins with interesting functions. SLC proteins mediate many essential physiological functions: they regulate lymphocyte signaling and control their differentiation, function, and fate by modulating diverse metabolic pathways and balanced levels of different metabolites [18]. In ID lymphocytes, a total of five SLC proteins showed a distinctly higher abundance after PWM stimulation: SLC1A5 (PWM/unstim ratio: 23.4), SLC38A1 (PWM/unstim ratio: 16.2), SLC1A4 (PWM/unstim ratio: 13.9), SLC29A1 (PWM/unstim ratio: 12.2), and SLC3A2 (PWM/unstim ratio: 7.5; all Table 1, light blue; Table S2). SLC1A5 and SLC38A1 co-transport polarized Na^+^ and glutamine, while concentrated glutamine is exchanged for leucine by the SLC7A5–SLC3A2 complex [18]. Glutamine and leucine promote the activation of mTORC1, which regulates T cell metabolism and cell differentiation of the Th1 and Th17 subsets [18]. Thus, the increase of SLC protein abundances indicates a very strong T cell reaction of ID lymphocytes to the PWM stimulus and may point to the preferred T helper pathway of ID lymphocytes after stimulation. 

CD200 was 6.9-times more abundant in ID lymphocytes after PWM stimulation (Table 1, blue; Table S2). It is expressed in activated T lymphocytes, B lymphocytes, thymocytes, neurons, and endothelial cells [41,42,43], while CD200 receptor expression is restricted to specific T lymphocyte subpopulations and myeloid cells [44,45]. The CD200 receptor activation by CD200 results in the suppression of the inflammatory response of macrophages and cytotoxic T lymphocytes, which activates regulatory T lymphocytes (Treg) and induces tolerance [46,47]. Zhu et al. found that in mouse macrophages, CD200 leads to a significant suppression of the *S. aureus*-induced production of proinflammatory cytokines and nitric oxide [48]. Concomitantly, the deletion of CD200 boosted the bactericidal capability of the mouse macrophages [48]. Therefore, the distinctly higher expression of CD200 in ID lymphocytes after immune stimulation points to an immunosuppressive impact on the immune reaction of those cows. 

A very strong increase of abundance in lymphocytes of aberrant vaccination responders was detectable for CD83. After stimulation with PWM, CD83 abundance rose by 86.8-fold compared to unstimulated ID lymphocytes (Table 1, light blue; Table S2), and also in Western blot analysis, the higher CD83 expression after PWM stimulation was verified in PBMC of further ID animals (Figure 2B). To our knowledge, the expression pattern of CD83 on cow blood cells has not been further characterized. In mice, CD83 is expressed on activated immune cells, including T and B cells, monocytes, neutrophils, microglia, and dendritic cells [49,50], and it plays a major role in resolving and controlling immune responses [49]. CD83 is a crucial factor during the differentiation of B and T cells and the development and continuation of tolerance [49], as it is essential for the differentiation and stability of Tregs [51]. Thus, CD83 deficiency caused strongly decreased numbers of differentiated FOXP3^+^ Tregs in Treg-specific CD83 knock-out mice [51]. In these mice, typical differentiation markers for Tregs like IL2RA were found to be downregulated, while proinflammatory cytokines were upregulated [51]. Overall, Treg-specific CD83 knock-out mice exhibited an impaired tolerance [49]. Thus, the strongly increased abundance of CD83 in lymphocytes of ID cows after PWM stimulation could point to the differentiation of Tregs and a distinct tolerance of those ID cows after such immune stimulation. 

IL2 and its ligand IL2RA are essential to immune homeostasis and lymphocyte differentiation [52,53]. Depending on the antigenic stimulus and the prevalent cytokine milieu, IL2 regulates lineage commitment of different CD4^+^ Th cell subsets [54]. Furthermore, IL2 signaling via IL2RA plays an important role during Treg differentiation, expansion, and function [55]. Therefore, it is of particular interest that the IL2RA abundance in ID lymphocytes was strongly increased after PWM stimulation (PWM/unstim ratio: 30.0; Table 1, light blue; Table S2) and that this significant increase of IL2RA expression could also be confirmed in further ID animals by the Western blot analysis (Figure 2A). While IL2RA is constitutively expressed in human CD4^+^ Treg cells (FOXP3^+^ CD25^+^) [56], in other activated CD4^+^ T cells, IL2RA transcription is strongly induced by antigenic or mitogenic stimuli [57]. Thus, besides the possibility that the increased IL2RA expression of ID lymphocytes originates from Tregs, another explanation could be the mitogenic stimulation. After mitogenic stimulation, IL2RA transcription is triggered by a TCR-CD3 engagement, and co-stimulatory signals are mainly induced by CD28 [57]. This T cell priming activates several transcription factors [57], which increase the expression of cell surface receptors, including IL2RA, and of multiple cytokines, including IL2 [58]. Vice versa, IL2 expression activates IL2RA transcription [59]. Remarkably, in lymphocytes of aberrant vaccination responders, CD28 as well as IL2 abundance were both increased after PWM stimulation, underlining the use of this immune pathway. CD28 abundance was 1.9-times enhanced (Table S1), which is of particular interest, as ID lymphocytes also strongly hyperproliferate upon in vitro stimulation with ConA [5]. In mice, this mitogen causes mainly T cell stimulation via CD28 through co-stimulation of CD80 and CD86 [60]. IL2 abundance rose 30.5-times through mitogenic stimulation in ID lymphocytes but also 17.3-times in control lymphocytes (Table 2, light blue; Table S5), which can be explained by the IL2 dependency of the PWM-induced T cell stimulation [22]. Interestingly, we recently showed that IL2 stimulation in vitro also leads to a highly significant (*p* > 0.0001) hyperproliferation of ID lymphocytes compared to the lymphocytes of control cows [5], which also highlights the importance of this axis for the immune deviant phenotype. These data also raise the possibility that both immunophenotypes respond in the same direction to the PWM stimulus but that the ID cows respond significantly more strongly and exuberantly. Furthermore, earlier, we detected an enhanced expression of the signal transducer and activator of transcription (STAT) 3 and 5A in ID lymphocytes after PWM stimulation [5]. Since phosphorylation of the IL2 receptor coupled to JAK tyrosine kinases permits the recruitment of STAT5A, STAT5B, and STAT3 [61], our data clearly demonstrate downstream signaling of the IL2 receptor in ID lymphocytes after PWM stimulation. Due to the fact that STAT3 as well as STAT5 can induce different Th subsets [62], no conclusions regarding the preferred T helper reaction by ID lymphocytes after PWM stimulation could be drawn yet. 

Curiously, the protein with the strongest increase of abundance after PWM stimulation in ID lymphocytes was IFNγ (PWM/unstim ratio: 177.6; Table 2, light blue; Table S5). IFNγ is well described for its Th1 response: via STAT1, it induces T-bet, the master transcription factor for Th1 cells, which in turn support IFNγ production in a feed-forward manner [63]. However, there is also emerging evidence that CD4^+^ FOXP3^+^ Treg cells can produce the proinflammatory cytokine IFNγ when stimulated in a Th1 cytokine environment [64]. A study by Koenecke et al. reported that murine FOXP3^+^ Treg cells readily produced IFNγ in vivo in a highly inflammatory model of graft-versus-host disease and during a Th1-dominated immune response to intracellular bacteria [64]. This indicates that Treg cell-intrinsic IFNγ production is required for their protective function [64]. The authors reasoned that IFNγ produced by FOXP3^+^ Treg cells has essential immunosuppressive functions that are required for the prevention of experimental graft-versus-host disease, which hints at a more universal function of this cytokine beyond its role in classical Th1-type immune responses [64]. 

Summed up, many important immunological key players are higher abundant in ID lymphocytes after immune stimulation with PWM. Some data suggest the hypothesis of an immunosuppressive Treg differentiation after immune stimulation in those cows responding aberrantly to PregSure BVD vaccination and, respectively, the possibility of a same-directed but significantly stronger immune response compared to control cows. Since it has become clear in recent years that not only one type of T helper response is often developed in the course of an infection but that several responses can alternate [65], it must also be considered that several immune responses to the PWM stimulus are developed. Further studies must follow to clarify this. 

In a next step, we investigated whether the immune phenotypes react functionally differently by analyzing the totality of the respective altered proteins. Therefore, we performed pathway enrichment analyses using exclusively more abundant proteins (PWM/unstim ratio ≥ 2) in either control or ID samples. Clear differences in the pathway enrichment between the control and ID cows became evident (Figure 3 and Figure 5). The comparison for the cellular dataset revealed many biosynthetic processes and apoptotic signaling pathways over-represented in the control lymphocytes after PWM stimulation (Figure 3A, Table S3). Contrarily, in ID cells, one of the most enriched pathways was “viral process” (Figure 3B, Table S3). Noticeably, an additional analysis of pathways associated to proteins more abundant in both the control and ID lymphocytes after PWM stimulation revealed IL2RA as well as CD83 in the pathway for “response to stress” (Table S3). Focusing on the pathway enrichment analysis of the secretome dataset, the most over-represented pathways in control cows could be associated to the immune response to the stimulus (Figure 5A, Table S6). On the other hand, the GO terms to the most over-represented pathways in ID cows were rather linked to protein biosynthesis (Figure 5B, Table S6). The reason for this significant deviation is the large number of more abundant ribosomal proteins in ID cells after PWM stimulation. Thus, besides important immunological key players, 45% of the more abundant serum proteins after PWM stimulation in ID cows belong to the family of ribosomal proteins (Table S6). Although ribosomal proteins are essential for protein translation and ribosome assembly, they also possess ribosome-independent functions that have been recognized in the past [19]. Disruption of the ribosome biogenesis by various intra- or extracellular stimulations results in ribosomal stress that leads to the accumulation of a ribosome-free form of ribosomal proteins [66]. Those ribosome-free ribosomal proteins can enrich in the nucleoplasm, where they are able to fulfil their ribosome-independent functions, like immune signaling as well as tumor promotion and suppression [19]. The cause for ribosomal stress can be, amongst others, chemical agents [19]. Thus, it seems possible that the ID lymphocytes entered ribosomal stress as a result of stimulation with PWM and following hyperproliferation, which the control lymphocytes apparently did not, or at least not to this extent. Some of the differentially more abundant ribosomal proteins (RP) in ID lymphocytes after PWM stimulation were associated with a positive regulation on different viral infections [67]. Thus, RPL18 (Table 2, blue; Table S5) was amongst other features already described to promote and be required for viral translation and replication [68,69], as well as to enhance viral replication [70]. RPL6 (Table 2, blue; Table S5) was featured to facilitate the production of viral particles of *Human T-cell leukemia virus type 1* [71], and RPL7 (Table 2, blue; Table S5) is described as contributing to the development of *Human immunodeficiency virus type 1* infections [72] as well as of *White spot syndrome virus* in fish [73]. These pathway enrichment analysis data indicate a kind of misdirected immune reaction of ID lymphocytes after mitogenic stimulation with PWM. 

Limitations of this study are that no proteoforms of the identified proteins could be quantified due to the peptide-centered bottom-up approach used in this study. The possible effect of the proteoforms of the identified proteins should therefore be functionally validated in further studies. Beyond that, the small sample size in the proteomic datasets could be another limitation of this study. However, since we examined all PregSure BVD vaccinated cows available to us at the time and these are now no longer alive, it was not possible for us to increase the number of animals examined in the discovery proteomic approaches. Therefore, Western blot experiments with a larger sample size were performed to validate our results. The data on ID cows examined in the mass spectrometric analyses were exclusively from cows that had shown a pathological response to vaccination with PregSure BVD by producing BNP-triggering alloantibodies. However, the Western blot analyses of IL2RA and CD83 expression (Figure 2) clearly confirmed that lymphocytes from PregSure BVD unvaccinated ID cows also responded in such a deviant manner to immune stimulation with PWM. Furthermore, since 22% of 121 Pregsure BVD unvaccinated cows elaborately examined in in vitro proliferation assays also reacted just as significantly hyperproliferatively to various polyclonal stimulants as the Pregsure BVD vaccinated BNP cows [5], it can be assumed that the ID cows that were not vaccinated with PregSure BVD would develop a similar deviating immune response upon respective vaccination. In addition, we see the possibility of undesirable vaccination reactions from ID cows in future vaccinations, because the vaccination meets an immunologically different host that reacts unexpectedly. We also consider that the immune response of ID cows, even if significantly different from that of control animals, may be just as effective in certain issues, depending on the triggering immune stimulus. There is also a need for further research here, as well as on the question of whether the ID phenotype is inherited or acquired.

Overall, it is interesting that PBMC of cows from one dairy farm, which had the same breed and were similar in age and parity, showed differentially abundant proteins in response to the same stimulus to that extent, which indicates the development of completely different immune responses. Further experiments are required to identify the exact pathways. In any case, the data are of great importance for the cow population, as the practical relevance of the immune deviant phenotype was already evident from the pathological response of ID cows to vaccination with PregSure BVD. Hence, it has already been proven that this ID phenotype can show functional effects. There is thus a great risk that this immune phenotype will react pathologically again in the case of other vaccinations, as well as in the case of a natural infection. Furthermore, as cows are food-producing animals, there is a potential threat to food safety from the deviant handling of pathogens by ID cows.

## 5. Conclusions

The entity of differentially abundant proteins between control and ID cows before and after stimulation with PWM underlines the divergent reaction of ID cows to immune stimulation and, therefore, gives a possible explanation for the pathological response to the vaccination with PregSure BVD. Our study provides a comprehensive in-depth dataset for the lymphocyte proteome of cows with different immune phenotypes and reveals new insights into proteome changes after polyclonal stimulation with PWM. The data consolidated the deviant immune reaction of the bovine ID phenotype to an immune stimulus that might also occur in vivo after vaccinations or natural infections. The highly different abundance of CD83 in the ID animals highlights the importance of further investigations into this molecule in the bovine immune response. Moreover, the present study gives not only basic information about immune responses from cows, but it is also important for the understanding of pathophysiology, e.g., in adverse immune responses to vaccinations, as seen with fatal BNP. 

## Figures and Tables

**Table 1:** Human orthologue gene names of differentially abundant proteins after PWM stimulation in PBMC of bovine immune phenotypes. Significantly (*p* ≤ 0.05) differential proteins (ratio ≥ 2) in peripheral lymphocytes of control (*n* = 2) and ID cows (*n* = 2) after in vitro stimulation with PWM. In control lymphocytes (white header and light blue header), 134 proteins were differentially more abundant after the PWM compared to unstimulated cells. In lymphocytes of ID cows (blue header and light blue header), 166 proteins were differentially upregulated after in vitro stimulation. Thereof, 107 proteins were identified in both control and ID lymphocytes (light blue header). Proteins that are referred to in the text are in bold.

Protein Abundance per Phenotype	Up in Controls	Up in Controls and Immune Deviant Cows				Up in Immune Deviant Cows	
No. of proteins	**27**	**107**				**59**	
Gene names	CACYBP	KPNA2	ISG20	GBP4	CARS1	**CD200**	DPP4
	LAP3	GBP6	MCM6	TNFSF8	IARS1	RFC5	IPO5
	ADK	CBS	IFI44L	**SLC1A5**	**SLC38A1**	IPO4	ABCF1
	**LRRC59**	MTHFD2	NOC3L	AASDHPPT	PCNA	SEMA4A	HAT1
	**CLIC4**	PYCR1	AARS1	EED	RPL23	UTP3	ALDH18A1
	PPAT	PABPC4	DNMT1	**SLC3A2**	TARS1	NAT10	EIF4A1
	H1-2	G3BP1	PCK2	MDN1	ATP1B3	DDX21	AHSA1
	U2AF1	PSAT1	RPL23	**SLC1A4**	TBCD	RFC4	TF
	H1-1	TNFRSF4	UBA7	RAP1A	RPL27A	LRPPRC	ABCE1
	ELAVL1	RPL37A	PSPH	P2RX7	RPL27	FAS	GPR183
	HNRNPA1	GBP1	CCT6A	SLAMF1	CAD	GTPBP1	MGAT2
	SYNCRIP	WARS1	RRM1	ICAM3	FNBP1	NOP2	DDX3X
	NAMPT	GBP6	MCM7	STAT3	ACAD10	HPF1	HSPD1
	TIMM50	UHRF1	PAF1	SHMT2	NUDC	GNE	YARS1
	OLA1	HERC6	B2M	PYCR2	EIF4G1	HLA-E	RPL8
	CPOX	PPA1	SMC4	ACOT7	**SLC29A1**	RPL3	GSPT1
	RALY	RPS10	HSP90AB1	UBA5	DOCK10	TMEM87A	JAML
	IMPDH2	MCM4	BCAT1	CD69		STX11	PMPCB
	HARS1	MTHFD1L	ICAM1	ALCAM		TFRC	PHGDH
	TRAP1	G3BP2	SMC2	EIF3H		GMPS	ATAD3A
	HYOU1	ISG15	GARS1	MOV10		HLA-E	NMT1
	PARK7	ASNS	CDK1	POLD1		PPID	SWAP70
	RPL36	NUB1	**IL2RA**	FKBP4		TSR1	RPS27
	PNP	MCM5	**CD83**	RPL21		BSG	IPO7
	MX1	FDPS	DNAJA1	LARS1		EIF5	HLA-E
	PABPC1	MCM2	MCM3	ABCB4		CCDC47	KTN1
	NAA50	GBP1	TIAL1	EIF5B		RPL35	CD44
		DHX30	EIF4G2	EEF1D		HSPH1	RPS8
		GBP4	IFI44	RPS7		MTHFD1	RPS23
		EIF2S2	TNFRSF18	ICOS		RPL15	

**Table 2:** Human orthologue gene names of differentially abundant proteins after PWM stimulation in PBMC secretome of bovine immune phenotypes. Differential proteins (ratio ≥ 2) in secretomes after in vitro PWM stimulation of PBMC from control (*n* = 1) and ID cows (*n* = 1). In the secretome of control lymphocytes (white header and light blue header), 38 proteins were differentially more abundant after PWM compared to the secretome of unstimulated cells. In the secretome of ID cows (blue header and light blue header), 47 proteins were differentially more abundant after in vitro stimulation. Thereof, 16 proteins were identified in both control and ID lymphocytes (light blue header). Proteins that are referred to in the text are in bold.

Protein Abundance per Phenotype	Up in Controls		Up in Controls and Immune Deviant Cows	Up in Immune Deviant Cows	
No. of proteins	**22**		**16**	**31**	
Gene names	MMP1	**IL17F**	**CCL5**	RPS7	OAS1
	**IL17A**	LYZ	INHBA	RPL3	**RPL18**
	MMP3	IL1RN	**IL2**	SF3B1	**RPL6**
	**CXCL8**	EIF4G1	GBP1	NUCB1	RPL30
	CXCL1	LCN2	IDO1	PRMT1	**RPL7A**
	CXCL2	SSB	GZMA	RPL5	MVP
	CAMP		TGM2	CTSB	HNRNPR
	HBA1		NOS2	HNRNPM	SYNCRIP
	PGLYRP1		ICAM1	XDH	ARPC3
	ARMC10		**IFNG**	AARS1	SULT1A1
	CA2		PPA1	RPL18A	HNRNPA3
	HBB		WARS1	RPS8	RPL14
	SLC4A1		LTF	RPS5	CORO7
	CAMP		PTX3	RPS9	RPS10
	UPP1		SDS	EIF4A1	MACROH2A1
	GLG1		NUCB2	RPL27	

## Data Availability

The mass spectrometry proteomics data presented in this study have been deposited to the ProteomeXchange Consortium via the PRIDE partner repository (https://www.ebi.ac.uk/pride/ (accessed on 31 January 2020 for the cellular dataset and on 5 March 2020 for the secretome dataset) [15]. Data are openly available via identifiers PXD017350 (cellular dataset) and PXD017892 (secretome dataset). Additionally, the whole proteomics data presented in this study are available in Tables S1 and S4.

## Supplementary Materials

The following are available online at https://www.mdpi.com/article/10.3390/proteomes10010007/s1, Table S1: all identified proteins of the cellular dataset; Table S2: the identified proteins of the cellular dataset_ratio2 p005; Table S3: cellular data pathway enrichment analysis; Table S4: all identified proteins of the secretome dataset; Table S5: identified proteins of the secretome dataset_ratio2 p005; Table S6: secretome data pathway enrichment analysis; Figure S1: Western Blot membranes.
