# Supplementary material for: Bovine Peripheral Blood Derived Lymphocyte Proteome and Secretome Show Divergent Reaction of Bovine Immune Phenotypes after Stimulation with Pokeweed Mitogen
Source: Proteomes. 2022 Feb 8;10(1):7. doi: 10.3390/proteomes10010007 (PMC8883952; doi:10.3390/proteomes10010007)
Supplement: Supplementary file 1 [file proteomes-10-00007-s001.zip › Figure S1_Western Blot membranes_Kleinwort et al..pdf]

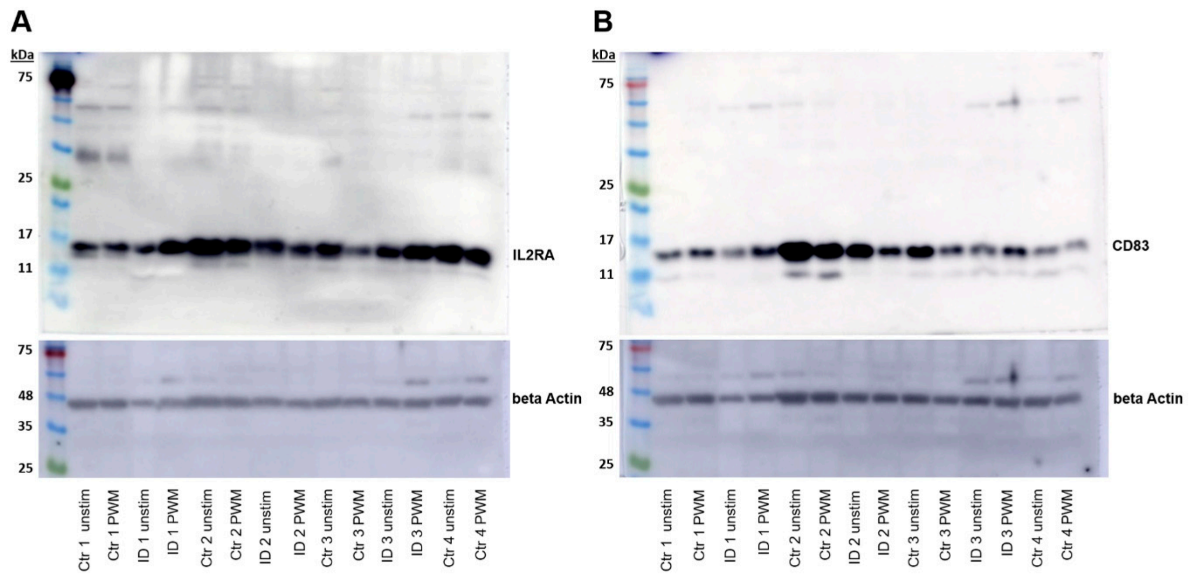

Figure S1. Representative western blots of bovine PBMC lysate for IL2RA (A) and CD83 (B). Reincubation with beta Actin (A and B) was performed for normalization.
